# Examining social norm impacts on obesity and eating behaviors among US school children based on agent-based model

**DOI:** 10.1186/1471-2458-14-923

**Published:** 2014-09-06

**Authors:** Youfa Wang, Hong Xue, Hsin-jen Chen, Takeru Igusa

**Affiliations:** Department of Epidemiology and Environmental Health, School of Public Health and Health Professions, University at Buffalo, State University of New York, Buffalo, NY USA; Johns Hopkins Global Center on Childhood Obesity, Bloomberg School of Public Health, Johns Hopkins University, Baltimore, USA; Institute of Public Health, National Yang-Ming University, Taipei, Taiwan; Department of Civil Engineering, Whiting School of Engineering, Johns Hopkins Systems Institute, Johns Hopkins University, Baltimore, USA

**Keywords:** Agent-based model, Body mass index, Child, Healthy body image, Obesity, Overweight, Social norms, Networks

## Abstract

**Background:**

Although the importance of social norms in affecting health behaviors is widely recognized, the current understanding of the social norm effects on obesity is limited due to data and methodology limitations. This study aims to use nontraditional innovative systems methods to examine: a) the effects of social norms on school children’s BMI growth and fruit and vegetable (FV) consumption, and b) the effects of misperceptions of social norms on US children’s BMI growth.

**Methods:**

We built an agent-based model (ABM) in a utility maximization framework and parameterized the model based on empirical longitudinal data collected in a US nationally representative study, the Early Childhood Longitudinal Study – Kindergarten Cohort (ECLS-K), to test potential mechanisms of social norm affecting children’s BMI growth and FV consumption.

**Results:**

Intraclass correlation coefficients (ICC) for BMI were 0.064-0.065, suggesting that children’s BMI were similar within each school. The correlation between observed and ABM-predicted BMI was 0.87, indicating the validity of our ABM. Our simulations suggested the follow-the-average social norm acts as an endogenous stabilizer, which automatically adjusts positive and negative deviance of an individual’s BMI from the group mean of a social network. One unit of BMI below the social average may lead to 0.025 unit increase in BMI per year for each child; asymmetrically, one unit of BMI above the social average, may only cause 0.015 unit of BMI reduction. Gender difference was apparent. Social norms have less impact on weight reduction among girls, and a greater impact promoting weight increase among boys. Our simulation also showed misperception of the social norm would push up the mean BMI and cause the distribution to be more skewed to the left. Our simulation results did not provide strong support for the role of social norms on FV consumption.

**Conclusions:**

Social norm influences US children’s BMI growth. High obesity prevalence will lead to a continuous increase in children’s BMI due to increased socially acceptable mean BMI. Interventions promoting healthy body image and desirable socially acceptable BMI should be implemented to control childhood obesity epidemic.

## Background

Although the importance of social norms in affecting health behaviors is widely recognized, the current understanding of the social norm effects on obesity is limited. The dynamic interplay between individuals, groups, and environment poses challenges to traditional methods to realize a holistic view of “contagious” obesity [1, 2].

There have been efforts to identify pathways and mechanisms in peer influences. Eisenberg et al. measured social norm effects by asking respondents whether they had friends who dieted to lose or keep from gaining weight [3]; Thompson et al. [4] used multifaceted scales to measure peer influence, such as the degree of teasing that respondents received from friends about weight and appearance, and their friends’ influence on their ideas of body image and weight-control strategies; Shomaker and Furman [5] measured the social reinforcement of thinness from close social group members (e.g., mother, close friends and romantic partners); Christakis and Fowler [1] examined obesity spreading through social networks using the Offspring Cohort from the Framingham Heart Study [1]. However, traditional analytical approaches have led to mixed empirical findings in the literature. The influence of a social network might disappear once other social and contextual factors were considered in the analytical models [2]. Different results came from the alternate foci and hypotheses based on a reductionist point of view.

Systems methods attempt to examine the research question as a whole. Agent-based models (ABM), as a major application of systems methods, simulate agent behaviors at the micro level and can generate macro level emergent patterns from the bottom up. With the aid of ABM, this study aimed to examine the dynamics between social norms and individual children’s body mass indices (BMI) and their consumption of fruits and vegetables (FV). We also tested how misperception of the social norm may affect children’s BMI growth. Based on rationality and assumption of utility maximization, a group of school children’s BMI and FV intake within social networks (schools) are simulated. Since social influence on individuals’ BMI is usually masked and mixed with food and built environment effects, there has been considerable debate about whether obesity is contagious. The results from our ABM simulations intend to answer this question from a generative standpoint.

## Methods

### Overview of study design

We developed an ABM based on longitudinal data collected from a US nationally representative study, the Early Childhood Longitudinal Study – Kindergarten Cohort (ECLS-K). The data we used included those on students’ BMI and FV consumption collected in the 5^th^ and 8^th^ grades. We conducted simulation analysis using the ABM.

### Agent-based model, a systems model

**Figure 1:**
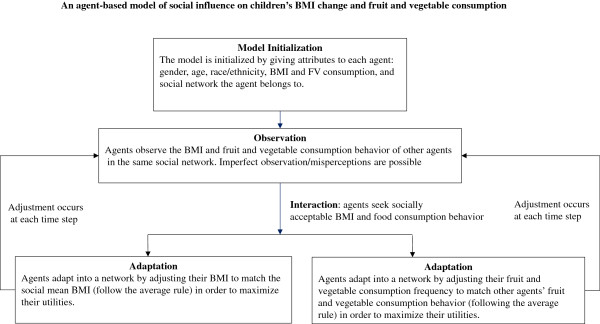
**An agent-based model (ABM) of social influence on children’s BMI change and fruit and vegetable consumption.**

We built an ABM to examine and test the possible mechanisms of social norm effect on children’s BMI and fruit and vegetable consumption behavior. In our model, the agents are children. The children interact with each other in a given social network which is the school they attend. The objective of each child is to maximize his/her utility. All the children observe the socially acceptable body image (quantified by BMI in the model) and the consumption behavior in their network. Under the social norm influence, deviation from the socially acceptable body image and consumption behavior causes disutility. These are the rules govern how these children interact with each other and how the interaction leads to the self-adjustment of children’s BMI and FV consumption. Figure 1 describes the structure of our ABM.

### Behavioral rules for the ABM

The key task of our ABM is to assess the social norm effect. There is no common definition for social norms. In a general sense, social norms are rules that guide individual behavior in interactions with others. Group pressure ensures the avoidance of the deviant acts in group members’ behavioral decision-making process. The norms and how individuals communicate the norms play a central role in the process. In our model, social norm effect was modeled through the behavior rule that governed children’s interactions, i.e. the follow-the-average (FTA) rule [6]. The rule defines how individuals communicate with the social environment. Individuals evaluate their status and adjust to engage in socially acceptable behaviors. The reference group where the normative information is derived from is a determinant of the communication and interaction patterns as well. The following sections explain how the behavior rule was defined in the ABM to capture the social norm effect.

#### The effect of social norm on children’s BMI

Each agent (child) exists within a social network (i.e. a school), and is subject to equal peer influence within that network. Agents interact with each other, obeying the follow-the-average rule in which agents adjust their BMI to match the mean BMI in their social network. Thus, within a social network, everyone contributed to the construct of the social norm and was influenced by it. The boundary of the social network was assumed not to extend beyond the school that an agent attends, i.e. no communication or spill-over effect between schools. At baseline, each agent was assigned an initial BMI according to the empirical data.

Under the FTA, the BMI for agent *i* at time *t* can be expressed in a simple form:
1

where DIF is the difference between the individual BMI and the social mean BMI at the beginning of the last time interval, *E* captures the average growth trend as children age, as determined by genetic and biological factors, and shared environmental factors. *α* is the average net effect of social norm on BMI change. The heterogeneity was explicitly introduced by a random shock drawn from the uniform distribution in the range between 0 and 1. This shock modified the average net social norm effect across individuals and was used to represent an individual’s capability to adjust to a socially acceptable BMI level given restrictions imposed by physical, psychological, environmental, and other contextual factors.

#### The effect of social norm on children’s FV consumption

Similarly, agents’ FV consumption behavior was defined as:
2

where DIFV is the difference between the individual FV consumption and the social mean FV consumption at the beginning of the last time interval, *FV*_*it-*1_ is the FV consumption index of agent *i* at time *t-1*, *FV*_*it*_ is the FV consumption index of agent *i* at time *t*, β is the average propensity for agents to adjust his/her FV consumption to fill the gap, and *δ* is a random draw from a standard unit normal random variable, representing the individual heterogeneity due to environmental, physiological, psychological, and other unobservable factors.

#### Hypothetical scenarios of misperception of the social norm

In our model, we assumed that children can perfectly observe the social mean BMI and fruit and vegetable consumption behavior. However, in reality, imperfect observation may occur, which leads to misperception of the social norm. Based on the learned parameters in the previous model settings, we examined the impact of misperceptions on children’s BMI changes. We constructed three hypothetical scenarios in which follow-the-average remains as the governing rule, but children with a BMI above social average may wrongly perceive the real situation:

- Scenario 1. Qualitative deviance misperception, i.e. children with a BMI above real social average perceived their BMI as under the average and make opposite BMI adjustments.
- Scenario 2. Consensus misperception, i.e. children with a BMI above real social average perceived others are having similar BMI and make no attempt to make changes.
- Scenario 3. Probabilistic quantitative misperception, i.e. children with a BMI above social average correctly observed the real social average BMI with a range of probabilities. For illustration purpose, we assumed 50% of these children are able to make the expected changes while the remaining 50% follow Scenario 1.

### Calibration and validation of ABM generated findings

#### Model calibration and validation

From the generative perspective, the validity of an ABM crucially depends on how well the model-generated data can reproduce the observed data. To our knowledge, there is no established algorithm available in literature to guide numerical parameter calibration for ABMs. We used the ECLS-K data to calibrate and validate our model. First, we initialized the model by assigning baseline attributes to each agent. These attributes included the gender, age, race/ethnicity, baseline BMI and FV consumption, and the social network the agent resided. The social networks had no spatial relationship, as we did not examine the spillover effect. The parameters to be estimated were α and β defined in the behavioral rules as described in the previous section. Then the model searches for the numeric values of α and β until the model generated data matched the empirical data to the greatest extent possible. That is, the information of the children at the 5^th^ grade was used for initial model setup, and the model then generated simulations for three years consequently, i.e. BMI and FV consumption in the 6^th^, 7^th^, and 8^th^ grade.

The criteria we used in our calibration and validation was the deviance of the descriptive statistics between the observed and the model-generated data distributions, i.e. deciles, the means, and the standard deviations. We compared the agreement between the observed and the ABM-generated data using moments, correlation coefficients, and distributional characteristics.

Our ABMs were designed using NetLogo 5.0 [7]. Other statistical analyses were conducted using SAS 9.2 and STATA 11.

#### Empirical data (Early Childhood Longitudinal Study – Kindergarten Cohort, ECLS-K)

As stated above, we used the ECLS-K data to obtain empirical parameters for our ABM. The ECLS-K is a study following a national representative sample of children recruited from 1280 schools across the US in 1998–1999 up to their 8^th^ grade (in 2007). Schools were the clusters for sampling and recruitment. Since food consumption frequencies were collected only at the follow-ups at the 5^th^ and 8^th^ grades, we utilized data from these two waves of follow-up for the current model: the 5^th^ grade as baseline and the 8^th^ grade as follow-up.

In this study, we selected children who stayed at the same school from 5^th^ to 8^th^ grade. We excluded those who transferred to another school to prevent the contamination of different schools in the model. Schools with 10 or more student participants in the 5^th^ grade were included in the analysis. Table 1 presents the summary characteristics of the eligible children in the data.

**Table 1:** **Distribution of the BMI and fruit and vegetable (FV) consumption of the ECLS-K children at 5** ^**th**^ **and 8** ^**th**^ **grade**

			Baseline at 5th grade				Follow-up at 8th grade			
Variables	Number of schools ^1^	Observations ^1^	Mean	SD	Min	Max	Mean	SD	Min	Max
BMI (kg/m^2^)	60	740	20.2	4.5	12.2	57.5	22.4	4.8	14.7	46.3
FV consumption (times/week)	60	740	18.6	17.2	0.0	115.5	19.2	14.0	0.0	86.0

^1^Number of schools and observations have been rounded for confidentiality purposes.

At each follow-up, the children’s body weight and height were directly measured twice. We used the averaged measurements to calculate BMI for each child. The 2000 CDC Growth Chart was used to convert children’s BMI into sex-age specific Z score and percentiles. The age-sex-specific percentiles of ≥85th and ≥95th were used to define overweight/obesity and obesity status, respectively [8]. To generate mutually exclusive weight status groups when examining the agreement between observed data and the ABM-simulated data, we defined non-overweight group as sex-specific percentiles <85^th^ or ≥ 95^th^, and non-obese group as sex-specific percentiles < 95^th^.

ECLS-K assessed children’s food consumption at the 5th and 8th grades. The FV index is a compound index constructed from children’s self-report consumption frequencies in the past week (7 days) of salad, potatoes, carrots, other vegetables and fruits. Children reported their consumption of these food items as zero, 1–3 times in last week, 4 to 6 times in last week, 1 time per day, 2 times per day, 3 times per day, and 4 or more times per day. We constructed a VF index score, a continuous variable, by adding up the separate consumption frequencies. We also categorized FV consumption as <3 vs. ≥3 times per day.

Please see the Appendix for detailed model documentation.

## Results

### Contribution of shared social and built environmental factors on concordance of US school children’s BMI and VF intake based on ECLS-K data

Table 2 shows the intra-class correlation coefficient (ICC) for BMI at 5^th^ grade was 0.064 and at 8^th^ grade it was 0.065, suggesting that there was similarity between children’s BMI within the same school. In contrast, ICC for VF dropped from 0.041 at 5^th^ grade to 0.034 at the 8^th^ grade, indicating diverging dietary patterns among older children. However, it is challenging to attribute the intra-school correlation to social vs. shared built environmental factors. The ABM simulation tends to add direct evidence about the social norm effect on children’s weight status and food behavior.

**Table 2:** **Observed and ABM-Predicted BMI and fruit and vegetable (FV) intake distributions at 8** ^**th**^ **grade**

		Intra-class correlation coefficient (ICC)	
Variables	Baseline (5th grade)	8 ^th^ grade	
		Observed	ABM-predicted
BMI (kg/m^2^)	0.064	0.065	0.067
FV consumption (times/week)	0.041	0.034	0.114

### ABM simulation findings on the effect of social norm on children’s BMI

After a thorough calibration and validation process as described in the previous section, we found that α for those whose BMI was below social average was 0.025 (kg/m^2^/year), while α for those whose BMI was above social average was 0.015. The general BMI growth trend *E* was found to be 0.65 kg/m^2^/year. Table 3 reports the descriptive statistic comparisons. It is evident that the ABM-simulated BMI distribution “mimics” well the observed BMI distribution. The correlation between simulated BMI and observed BMI was as high as 0.87. The mean squared error (MSE) between the simulation and the observation for BMI was 4.86^2^. After we silenced the social norm effect with growth trend unchanged, the MSE increased to 4.92^2^, indicating the existence of the social norm effects and the validity of the model, though the magnitude is small.

**Table 3:** **Observed and ABM-Predicted BMI (kg/m** ^**2**^ **) and fruits and vegetables (FV) intake distributions at 8** ^**th**^ **grade**

			Percentiles									
	Mean	SD	10	20	30	40	50	60	70	80	90	Correlation coefficient ^1^
**BMI (kg/m** ^**2**^ **) distribution at 8th grade**												
Boys and girls												
Observed	22.4	4.8	17.8	18.7	19.6	20.4	21.3	22.1	23.4	25.2	28.9	
ABM-predicted	22.2	4.4	17.9	18.7	19.4	20.2	21.1	22.2	23.6	25.3	27.8	
												0.9
Boys												
Observed	22.3	4.5	17.6	18.6	19.4	20.3	21.4	22.3	23.6	25.9	28.9	
ABM-predicted	22.2	4.1	17.7	18.5	19.3	20.1	21.4	22.7	24.1	25.8	28.2	
												0.9
Girls												
Observed	22.4	5.0	17.9	18.9	19.8	20.4	21.1	22.0	23.1	24.8	28.9	
ABM-predicted	22.2	4.7	18.0	18.9	19.5	20.4	20.9	21.9	23.3	24.6	27.2	
												0.9
**FV consumption (times/week) distribution at 8th grade**												
Observed	19.2	14.0	6	8	10	12.5	14.5	18	23	28.5	39	
ABM-predicted	19.7	14.8	8.0	10.0	11.3	12.8	14.4	17.5	21.0	26.9	37.7	
												0.3

^1^Correlation coefficient between observed and ABM -predicted values for BMI or FV.

**Figure 2:**
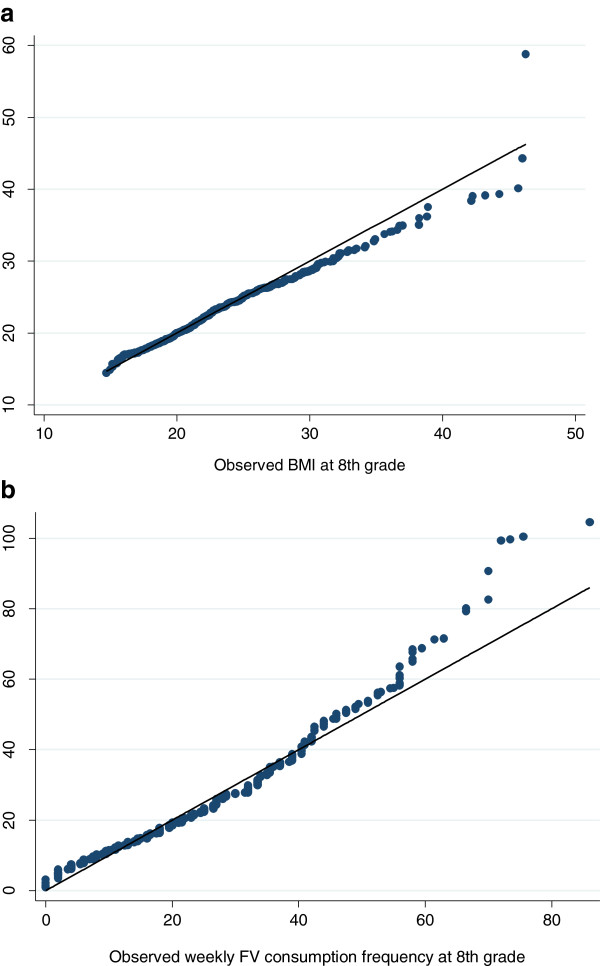
**Quantile-quantile (QQ) plot of US school children’s BMI and fruit and vegetable consumption distribution observed and ABM-predicted at 8th grade: ECLS-K data. a**. BMI distribution. **b**. FV consumption frequency distribution.

As Table 4 shows, we further categorized the children as normal, overweight, and obese based on the CDC 2000 growth chart. Kappa statistics suggested a high agreement between the observed and ABM-predicted weight status. Figure 2(a) shows a good agreement between the observed and ABM-predicted BMI distributions. These results suggest the validity of our hypothesized mechanism of the social norm effect on children’s BMI changes.

**Table 4:** **Agreement between observed and ABM-Predicted weight status and fruits and vegetables (FV) consumption for ECLS-K students at 8** ^**th**^ **grade**

Observed at 8th grade	ABM-predicted at 8th grade		
	N	N	Kappa ^1^
**Weight status**			
	Normal	Overweight/obese	
Normal	452	49	
Overweight/obese	40	195	0.72
	Non-overweight	Overweight	
Non-overweight	538	73	
Overweight	55	70	0.42
	Non-obese	Obese	
Non-obese	605	21	
Obese	30	80	0.72
**FV consumption**	< 3 times per day	> = 3 times per day	
< 3 times per day	378	102	
> = 3 times per day	138	118	0.26

^1^Kappa measures the agreement of between the observed and ABM -predicted values for BMI or FV.

Furthermore, the ICC based on observed and ABM-simulated data were also close: ICC for the observed 8^th^ grade BMI was 0.065, while the ICC for the ABM-simulated BMI was 0.067. The results suggest that the social norm added a motivational drive among the children, which generated an upward and converging trend of their BMI.

Gender difference was also apparent. The social norm was shown to have less impact on stimulating body weight reduction among girls and greater impact promoting weight increase among boys.

### ABM simulation findings on the effect of social norm on children’s FV consumption

The parameter for social norm effect was calibrated to be 0.14, while for children who consumed FV above the average level, the coefficient of social norm was calibrated to 0.05. Table 3 reports the summary statistics of the observed and simulated FV consumption distributions, showing that the ABM-predicted FV consumption under social norm influence was close to the observed, especially around the median. Figure 2(b) shows good agreement between the observed and ABM-predicted FV intake distributions.

Nevertheless, a comparison of the correlation coefficients between the observed and ABM-predicted VF intakes indicates that social norms might explain less FV consumption variance in our ABM. As Table 3 suggests, however, FTA did not perform well explaining FV consumption variations as evidenced by low Kappa statistics. This finding indicates that social norm may play a less influential role in children’s FV consumption.

### Simulation evidence on personal misperception and social consequences

According to the general model mentioned above, we compare the children’s BMI changes from 5^th^ grade to 8^th^ grade with and without the presence of misperceptions. Table 5 reports the mean BMI changes from 5^th^ grade to 8^th^ grade under the influence of misperceptions. It shows that individuals’ misperceptions of normative BMI would lead to an increase of overall mean BMI at group level. Among the three scenarios, the mean qualitative misperception would cause the largest mean BMI increase.

**Table 5:** **ABM simulation testing impact of misperception of social norm on social mean BMI changes- four scenarios** ^**1**^

	Baseline scenario	Scenario 1	Scenario 2	Scenario 2
		Qualitative deviance misperception	Consensus misperception	Probabilistic quantitative misperception
Grade	Mean	Mean	Mean	Mean
5th	20.15	20.15	20.15	20.15
6th	20.85	20.91	20.86	20.86
7th	21.52	21.64	21.55	21.54
8th	22.18	22.37	22.22	22.21

^1^Baseline scenario-- Individual children correctly perceive the social mean BMI and adjust accordingly. Scenario1. Qualitative deviance misperception, i.e. children with a BMI above real social average perceived their BMI as under the average and make opposite BMI adjustments; Scenario 2. Consensus misperception, i.e. children with a BMI above real social average perceived others are having similar BMI and make no attempt to make changes; and Scenario 3. Probabilistic quantitative misperception, i.e. children with a BMI above social average correctly observed the real social average BMI with a range of probability and 50% of these children are able to make the expected changes while the remaining 50% follow Scenario 1.

## Discussion

In the present study, using ABM and empirical data, we demonstrated the effects of social norms on US children’s BMI growth and FV consumption, i.e., a follow-the-average social interaction rule. We also showed how misperception of the social norm may affect children’s BMI growth. Our analysis showed a good agreement between observed and ABM- predicted BMI, e.g., the Pearson’s correlation coefficient was as high as 0.9, indicating the validity of our ABM, and the existence of the follow-the-average social norm effect. Furthermore, gender difference was apparent. The social norm was shown to have less impact stimulating weight reduction among girls and greater impact promoting weight increase among boys. Simulation experiments also showed that misperception of the social norm would further push up the mean BMI and make the distribution more skewed to the left. However, our simulation results did not provide strong support for the role of the social norm on students’ FV consumption.

### Social norms and children’s BMI changes

As the simulation results suggest, the influence of the social norm on children’s BMI adjustment is asymmetric, though the deviation from social average BMI could trigger the adjustment process in either case. It is more difficult for children to lower BMI growth than to gain BMI in order to meet the social norm. Moreover, the results suggest that for children whose BMIs were at the upper tail of the BMI distribution, their BMI adjustment behaviors become more heterogeneous and the social norm contributes little in explaining their BMI changes. This is evidenced by the relatively large difference between simulated and observed BMI for children above 70% in the BMI distribution. This phenomenon could be due to various endogenous and exogenous factors, which cause higher heterogeneous behavior among these children, for example, differences in energy metabolism and appetite control, and the influence of environment. Under-average children had a clear aim to attain growth above the average rate, while the over-average children faced the conflicting aims of fulfilling linear growth potential and preventing overweight/obesity. This dilemma adds to the complexity and heterogeneity of weight/BMI-change among the heavier children.

Our models also suggested gender differences in the social norm effect. The magnitude of weight increase influence was about 2 times higher than weight reduction influence among boys, while the ratio was about 7 times for girls. Possibly, girls may face more challenges than boys to lose weight due to factors related to dietary habits, physical activity level, and lifestyle. Furthermore, the results suggest that the social norm had greater impact on boys than girls with regard to weight increase. This result is in line with the finding of Bernier et al. that children in the lowest BMI percentile have a greater desire for change in body shape [9]. Difference in the perception and preference of body image across genders may partially contribute to the distinct patterns, for example, desire for a thinner or stronger body [10, 11].

### The social norm and FV consumption

The asymmetric social norm influence was evident in the FV consumption as well. The ABM simulation suggests that on one hand, children with low FV consumption may be influenced by the perceived social norm to increase their FV consumption; while those with high FV consumption may be less influenced by others in the same social network. Our findings indicate that the attributes of the FV, such as visual cues and palatability, could be more important than social norm when children are making food decisions, especially when their consumption reaches a threshold level [12]. Nutrition education about the health benefits of FV thus should intervene to facilitate desirable behavior changes. These results suggest the importance of nutrition education and the power of good role-models in initiating and maintaining high FV consumption.

The impact of the social norm on FV consumption was less evident than that on BMI adjustment. This is likely to be because low/high FV consumption may not cause immediate consequences in social binding and thus leads to less behavior changes. In contrast, body image can be directly observed and may incur negative psychological effects, such as suicidal attempt, lower self-esteem, and depression [13, 14]. Therefore, we may expect to observe much stronger social norm effects on children’s BMI than on their FV consumption behavior because of the difference in the seriousness of the consequences.

The weaker predicted or observed social norm impact on FV intake could also be affected by intra-individual variation in weekly food consumption. Moreover, FV consumption in the ECLS-K was measured in frequency, rather than amount. Although children consume a greater quantity of foods when growing up, the FV consumption pattern (frequency) may be established at earlier life stages. Hence, between 5^th^ and 8^th^ grades, children’s real FV consumption frequency might not change much with the FTA rule. Food environment and household dietary habits may have greater influence on children’s food choice than peer influence.

### Misperception and the social consequences

In general*,* the FTA rule governing social interactions and behavior changes crucially hinges on the assumption that every single member within a network perfectly observes the status and behavior of other members and thus is able to make correct assessment and behave accordingly. However, in reality, partially due to limited knowledge/information, the assessment is usually not based on comprehensive observation but rather on bounded information and subjective “guessing”. Our simulations suggest that the misperception would further push up the mean BMI and make the distribution more skewed to the left. From the intervention point of view, our findings imply that reducing misperception could potentially help prevent further increase in childhood obesity levels.

### Strengths and limitations

This study is the first ABM-based simulation study of the effects of social norms on childhood obesity. We tested the social norm impacts on BMI adjustment behavior and FV intake among children. We examined how misconception of the social norm can influence individual and social BMI outcome. Our model focused on the “individual-level effect”, rather than solely examining the weight distribution transitions. Furthermore, we added the time dimension in the analysis of the adjustment process. As an improvement to existing models, our model explicitly accounted for the heterogeneity of individuals and gender differences, and showed asymmetric social influence on children’s BMI. Burke and Heiland [14] used NHANES data to calibrate their model, but the data was too limited to identify social networks and analyze agent behavior within these networks [15]. The ECLS-K data we used provided natural well-defined social networks - the children’s schools - which largely enhanced the ability of our model to simulate real-world social norm effects.

Compared to the existing studies on the contagious nature of obesity among family members and friends, the ABM of this study overcomes the intrinsic limitations and gaps embedded in the traditional methods, such as the confounding effects of homophily and shared environment, mis-specified regression models and sensitive estimation approaches. Hypothetical mechanisms are explicitly simulated and tested through ABM, generating concrete evidence for us to make causal inference of the effect of social norms on the BMI distribution among children.

The study has a few limitations: First, the BMI model did not incorporate child’s behavioral approaches to adjust BMI under social norm influence. Changing energy intake and physical activity both can lead to BMI changes. We did not model specific behavioral changes during the BMI adjustment process. Second, due to the scope of this study, the model did not test the effectiveness of potential intervention options to promote desirable social norm effect and to inhibit negative impact simultaneously. Third, the model cannot distinguish pure social norm effects from the influence of other factors, such as the influence of the media and food environment at national and local levels, which could reinforce the social norm effect. We cannot develop a model that isolates the social norm effect due to data and methodological challenges, which deserves future research.

## Conclusions

In conclusion, our agent-based simulation analysis calibrated with empirical data demonstrated the effects of social norms on US children’s BMI growth, but was not clear on fruit and vegetable consumption. The follow-the-average social interaction rule could be one of the mechanisms that influence children’s BMI growth although it was not helpful in explaining fruit and vegetable consumption. These results indicate that health education and promotion of obesity awareness are important to prevent excessive weight gain in school children due to weight misperceptions. Moreover, the influence of social norms may differ by gender (boys are more likely to gain weight) and by baseline BMI. Hence, tailored strategies on different subpopulations may improve the efficacy of awareness promotion. Agent-based models can be useful to help explore social network factors affecting childhood obesity and eating behaviors.

## Appendix: model documentation

### Purpose

Social norm theory suggests that the behavior of individuals embedded in a social network is influenced by their perceptions of how other members believe and behave within a social system. Individual behavior is purposively rationalized to avoid disapproval of other group members. This model aims to examine: a) the effects of social norm on student’s BMI growth and fruits and vegetables (FV) consumption; and b) the effects of misperceptions of social norms on US children’s BMI growth.

### State variables and scales

We do not include spillover effects across social networks, so any relationship between schools are not modeled. The system reaches an equilibrium when all the sub networks reach their equilibria. State variables included in the models are:

1. BMI: Children’s body mass index
2. FV: Children’s weekly fruit and vegetable consumption frequency
3. Coordinates: X and Y coordinates of the schools representing the locations and boundaries of the social networks

### Process overview and scheduling

The model assumes rationality and utility maximization of agents. Deviation from socially determined mean body weight and eating behavior leads to disutility. An agent observes body images and eating decisions of other agents and adjust his/her own body weight and food intake to gain social acceptance and thus achieve their goals of utility maximization.

All of the agents have identical behavior rules. The adjustments are made at each time step. The updating occurs simultaneously for all the agents.

### Design Concepts

#### Emergence

The model explores the emergent phenomena of the patterns of fruit and vegetable consumption, BMI trajectory of the whole population, and trends in the prevalence of agents with BMI ≥85th and ≥95th percentiles of the CDC growth chart. These are the results that emerge from individual decisions of the agents and interactions between the agents.

#### Adaption

Agents in the system are allowed to learn, adjust, and update. They aim to adapt into a network by following the social mean behavior and weight status in order to maximize their utilities.

#### Interaction

Agents interact at local level. Agents interacted with each other by obeying the follow-the-average rule in which agents adjust their food intake and weight to match the mean BMI and fruit and vegetable consumption in their social network. Thus, within a social network, everyone contributed to the construct of the social norm and was influenced by it. The boundary of a social network was assumed not to extend beyond the school that an agent attends, i.e. there was no communication or spill-over effect between schools.

#### Sensing

Agents perfectly observe the BMI and fruit and vegetable consumption behavior of others in the same social network. Misperception scenarios in which information was not perfect were also examined.

#### Stochasticity

The heterogeneity of the agents’ ability to self-adjust was reflected by random draws from a uniform distribution.

#### Observation

Observations include BMI and fruit and vegetable consumption distributions and temporal trends.

### Initialization

The model is initialized by setting the attributes of the agents, including gender, age, race/ethnicity, and BMI and FV consumption at baseline and by assigning agents to specific social networks (schools) based on empirical data.

### Input

Further inputs are not required once the model is initialized.

### Submodels

Agents adjust their BMI to match the social mean BMI to maximize utilities. The interaction rule that governs BMI change for an agent *i* at time *t* can be expressed in a simple form:


where DIF is the difference between the individual BMI and the social mean BMI at the beginning of the last time interval, *E* captures the average growth trend as children age, as determined by genetic and biological factors, and shared environmental factors. *α* is the average net effect of social norm on BMI change. The heterogeneity was explicitly introduced by a random shock drawn from the uniform distribution in the range between 0 and 1. This shock modified the average net social norm effect across individuals and was used to represent an individual’s capability to adjust to a socially acceptable BMI level given restrictions imposed by physical, psychological, environmental, and other contextual factors.

Similarly, the interaction rule that governed agents’ FV consumption behavior was defined as:


where DIFV is the difference between the individual FV consumption and the social mean FV consumption at the beginning of the last time interval, *FV*_*it* − 1_ is the FV consumption index of agent *i* at time *t-1*, *FV*_*it*_ is the FV consumption index of agent *i* at time *t*, β is the average propensity for agents to adjust his/her FV consumption to fill the gap, and *δ* is a random draw from a standard unit normal random variable, representing the individual heterogeneity due to environmental, physiological and psychological factors.
